# Itinerários terapêuticos compartilhados por usuários de serviços especializados de saúde mental: uma análise por *clusters*

**DOI:** 10.1590/0102-311XPT052624

**Published:** 2025-01-13

**Authors:** Michelle Chanchetti Silva, Carlos Alberto dos Santos Treichel, Rosana Teresa Onocko-Campos

**Affiliations:** 1 Universidade Estadual de Campinas, Campinas, Brasil.; 2 Escola de Enfermagem, Universidade de São Paulo, São Paulo, Brasil.; 3 Faculdade de Ciências Médicas, Universidade Estadual de Campinas, Campinas, Brasil.

**Keywords:** Itinerário Terapêutico, Assistência à Saúde Mental, Serviços de Saúde Mental, Serviços Comunitários de Saúde Mental, Atenção Primária à Saúde, Therapeutic Itinerary, Mental Health Assistance, Mental Health Services, Community Mental Health Services, Primary Health Care, Ruta Terapéutica, Atención a la Salud Mental, Servicios de Salud Mental, Servicios Comunitarios de Salud Mental, Atención Primaria de Salud

## Abstract

Este estudo teve como objetivo identificar a existência de itinerários terapêuticos compartilhados por usuários de serviços especializados de saúde mental em um município de médio porte. Trata-se de um estudo transversal, realizado entre agosto e novembro de 2019 com 341 usuários de serviços especializados de saúde mental do Município de Itatiba, São Paulo, Brasil. Para identificação dos itinerários, a partir de um conjunto de variáveis, os usuários foram agrupados por meio da técnica de *cluster*. A melhor medida de silhueta de coesão e separação (> 0,3) para os *clusters* foi alcançada a partir de quatro variáveis: situação em que o problema de saúde mental foi identificado; local do primeiro atendimento; origem do encaminhamento para o serviço atual; e manutenção do vínculo com a atenção primária à saúde (APS). Os *clusters* identificados no estudo demonstraram: (1) baixa participação da APS no acolhimento dos casos novos com a maioria dos atendimentos acontecendo já em serviços especializados; (2) alta proporção de casos identificados em situações de crise; (3) baixa participação da APS na referência dos casos, com elevado acesso aos serviços especializados por meio de demanda espontânea; (4) falta de continuidade do cuidado na APS após a entrada no serviço especializado. Este estudo apontou para fragilidades importantes na rede de serviços estudada, evidenciando a necessidade de desenvolver estratégias que fomentem a integração dos serviços, especialmente no que se refere à APS, tanto para favorecer o acesso aos cuidados especializados em tempo oportuno como para viabilizar a continuidade do cuidado entre os diferentes pontos de atenção.

## Abstract (en)

This study aimed to identify the existence of therapeutic itineraries shared by users of specialized mental health services in a medium-sized municipality. This is a cross-sectional study, carried out from August to November 2019 including 341 users of specialized mental health services in the municipality of Itatiba, São Paulo State, Brazil. To identify the itineraries, based on a set of variables, the users were grouped with clustering. The best measure of silhouette of cohesion and separation (> 0.3) for the clusters was achieved based on four variables: situation in which the mental health problem was identified, place of first care, origin of referral to the current service, and bond maintenance with primary health care (PHC). The clusters identified in the study demonstrated: (1) low participation of PHC in welcoming new cases, with most of the care taking place in specialized services; (2) high proportion of cases identified in crisis situations; (3) low participation of PHC in the referral of cases, with high access to specialized services by spontaneous demand; (4) lack of continuity in PHC services after entering the specialized service. The study highlighted significant weaknesses in the studied healthcare network, evincing the need to develop strategies that foster the services integration, especially regarding PHC, both to favor access to specialized care in a timely manner and to enable the continuity of care between different healthcare facilities.

## Abstract (es)

El estudio tuvo como objetivo identificar la existencia de itinerarios terapéuticos compartidos por usuarios de servicios especializados de salud mental en un municipio de tamaño mediano. Se trata de un estudio transversal, realizado entre agosto y noviembre del 2019 con 341 usuarios de servicios especializados de salud mental del municipio de Itatiba, estado de São Paulo, Brasil. Para identificar los itinerarios, con base en un conjunto de variables, se agrupó a los usuarios mediante la técnica de clúster. La mejor medida de silueta de cohesión y separación (> 0,3) para los clústeres se logró con base en cuatro variables: situación en la que se identificó el problema de salud mental, ubicación de la primera atención, origen de derivación al servicio actual y mantenimiento del vínculo con la atención primaria de salud (APS). Los conglomerados identificados en el estudio demostraron (1) baja participación de la APS en la acogida de nuevos casos, y la mayoría de las atenciones ya se realiza en servicios especializados; (2) alta proporción de casos identificados en situaciones de crisis; (3) baja participación de la APS en la derivación de casos, con alto acceso a servicios especializados por demanda espontánea; (4) falta de continuidad del cuidado en APS tras el ingreso en el servicio especializado. El estudio señaló importantes debilidades en la red de servicios estudiada, poniendo de manifiesto la necesidad de desarrollar estrategias que fomenten la integración de servicios, especialmente en lo que respecta a la APS, tanto para favorecer el acceso oportuno a los cuidados especializados como para hacer viable la continuidad del cuidado entre diferentes puntos de atención.

## Introdução

De acordo com dados da Organização Mundial da Saúde (OMS), em 2019, aproximadamente um bilhão de pessoas conviviam com transtornos mentais em todo o mundo ^1^. No Brasil, estima-se que 6,9 milhões de brasileiros (3,3%) apresentem transtornos mentais graves e persistentes que requerem cuidados intensivos e contínuos, enquanto outros 30,2 milhões (14,5%) apresentam transtornos mentais leves ou moderados que requerem tratamento ocasional em serviços especializados ^2^.

Para viabilizar o cuidado a essa população, os movimentos de Reforma Psiquiátrica iniciados no Brasil na década de 1980 culminaram no estabelecimento de uma Política Nacional de Saúde Mental que pretende articular o cuidado em rede ^3^. Nesse sentido, a fim de garantir a articulação e a integração dos pontos de atenção, em 2011, por meio da *Portaria nº 3.088/2011*, foi instituída a Rede de Atenção Psicossocial (RAPS) ^3^.

Constituída por sete componentes distintos, mas interdependentes, a RAPS compreende os equipamentos de saúde que integram o Sistema Único de Saúde (SUS), nos variados níveis de complexidade, entre eles as Unidades Básicas de Saúde (UBS), Centros de Atenção Psicossocial (CAPS), serviços hospitalares, atenção de urgência e emergência, entre outros ^3^.

Apesar da importante demarcação política dessa estratégia de cuidado, aspectos como a falta de capacitação dos profissionais, especialmente nos serviços de atenção primária à saúde (APS) e a falta de delineamentos claros para referência e contrarreferência dos casos, além da escassez de recursos humanos e estruturais, têm atravessado a consolidação desse modelo de atenção, impactando diretamente no itinerário terapêutico das pessoas com transtornos mentais ^4^ ^,^ ^5^ ^,^ ^6^.

O itinerário terapêutico pode ser definido como o caminho que uma pessoa desenvolve em busca de auxílio para uma necessidade em saúde, o que inclui diferentes opções de cuidado, às vezes simultâneas, e a adesão ou o abandono do tratamento ^4^. A maneira como cada pessoa percorre essa trajetória em busca de saúde é singular, ancorada em suas vivências prévias e na forma como experimenta a doença de forma individual e coletiva. Especialmente no campo da saúde mental, além dos aspectos estruturais da rede de saúde, incluindo a oferta de serviços, essa experiência é atravessada pelos significados atribuídos à doença pelo indivíduo e por sua família, além do estigma social atribuído ao transtorno mental ^7^ ^,^ ^8^.

Os estudos sobre o itinerário terapêutico dos usuários têm um papel importante na compreensão sobre como os sistemas de saúde operam e quais momentos dessa trajetória necessitam de aprimoramento para a qualificação do acesso oportuno aos cuidados em saúde mental ^9^. Nesse sentido, estudos internacionais têm apontado para uma importante variação das formas de acesso e utilização dos serviços especializados de saúde mental, com diferenças significativas especialmente no que diz respeito ao tempo para acessar os serviços e ao papel da APS na identificação e encaminhamento dos casos ^7^ ^,^ ^8^ ^,^ ^10^.

Apesar dos avanços globais no estudo dos itinerários terapêuticos de pessoas com necessidade de cuidados em saúde mental, esse tipo de produção no Brasil ainda é incipiente ^9^. Uma revisão de literatura conduzida recentemente acerca dos caminhos para o cuidado em saúde mental no país apontou para uma escassez de dados, particularmente no que se refere aos arranjos utilizados para a atenção à crise e a integração entre os serviços hospitalares ou de urgência e emergência e os serviços comunitários ^4^.

Cabe ressaltar que a produção nacional acerca dos itinerários terapêuticos de pessoas com transtornos mentais se situa particularmente no campo qualitativo, havendo poucos estudos que tenham estudado as experiências coletivas de usuários de uma determinada rede de atenção ^4^. Nesse sentido, a fim de contribuir para a compreensão das experiências dos usuários na RAPS, este estudo tem como objetivo, a partir de uma abordagem de cluster, identificar a existência de itinerários terapêuticos compartilhados por usuários de serviços especializados de saúde mental em um município paulista de médio porte.

## Método

### Delineamento e participantes

Trata-se de um estudo transversal, realizado no período de agosto a novembro de 2019 com 386 usuários de serviços especializados de saúde mental de um município localizado no interior de São Paulo, Brasil. Sendo parte da Região Metropolitana de Campinas, o município contava com 120.858 habitantes ^11^ no ano em que o estudo foi desenvolvido. Sua RAPS era composta por 19 serviços de APS, além de um serviço hospitalar geral e outro de emergência, e três serviços especializados de saúde mental: Centro de Atenção Psicossocial II (CAPS II), Centro de Atenção Psicossocial - Álcool e Drogas (CAPS AD) e ambulatório de especialidades, frequentados por 1.958 usuários ^6^.

### Seleção dos participantes

A seleção dos participantes foi realizada por amostragem aleatória simples. A partir da lista de usuários dos serviços especializados foram selecionados os prontuários de 386 usuários que atenderam aos critérios de inclusão. Os usuários foram então contatados por telefone e convidados a participar do estudo. Os critérios de inclusão foram maiores de 18 anos e matriculados no serviço há pelo menos um mês. Foram excluídos do estudo os indivíduos com diagnóstico de deficiência intelectual, o que poderia comprometer sua capacidade de responder ao questionário.

### Procedimentos

A coleta de dados via questionários ocorreu no período de agosto a novembro de 2019, sendo realizada no próprio serviço em que os usuários estavam inseridos e em dias em que já tinham alguma outra atividade programada. Os questionários foram aplicados por um assistente social, uma médica e seis estudantes de psicologia que foram recrutados via processo seletivo e passaram por treinamento específico.

### Controle de qualidade e manejo dos dados

Os questionários aplicados com auxílio do software KoboToolbox (https://www.kobotoolbox.org) foram validados diariamente por uma supervisora de campo, com formação em psicologia e doutorado em saúde coletiva. Os dados foram baixados em formato Excel (https://products.office.com/), avaliados e corrigidos quando necessário e posteriormente convertidos para utilização no pacote estatístico Stata 16 (https://www.stata.com), software em que foram conduzidas as análises.

### Variáveis incluídas no estudo

As variáveis incluídas no estudo como preditoras do agrupamento (*cluster*) de usuários em torno de características comuns ao seu itinerário terapêutico foram: situação em que descobriu um problema de saúde mental (autodiagnóstico seguido de demanda espontânea; situação de crise; por sugestão de amigos ou familiares; consulta por queixas gerais); primeiro serviço em que recebeu atendimento para o problema de saúde mental (APS; serviços hospitalares ou de emergência; ambulatório de especialidade; CAPS II ou CAPS AD; serviços privados); local de encaminhamento para o serviço atual (demanda espontânea; APS; serviços hospitalares ou de emergência; ambulatório de especialidade; CAPS II ou CAPS AD; serviços privados); manutenção do vínculo com a APS (se recebeu ou não atendimento nos últimos seis meses).

Ademais, foram incluídas como variáveis independentes: sexo (feminino; masculino); cor (branca; parda; preta); idade, em anos (18-30; 31-45; 46-60; 61 ou mais); escolaridade, em anos de estudo (0-4; 5-8 anos; 9 anos ou mais); trabalho remunerado (sim; não); renda *per capita*, em salários mínimos (até ½; ½-1; mais de 1); diagnóstico (transtornos afetivos e neuróticos; transtornos psicóticos; uso problemático de substâncias psicoativas; ainda não diagnosticado).

### Análise estatística

Inicialmente, foi utilizada a estatística descritiva por meio da qual foram calculadas as médias das variáveis numéricas, bem como seus respectivos desvios-padrão, além do cálculo da prevalência de cada estrato das variáveis estudadas.

A partir das possíveis variáveis preditoras incluídas no estudo, o agrupamento dos usuários por *clusters* buscou a melhor medida de silhueta de coesão e separação possível, tendo-se obtido uma medida de > 0,3 a partir das variáveis: (1) situação em que descobriu um problema de saúde mental, (2) primeiro serviço em que recebeu atendimento para o problema de saúde mental, (3) origem do encaminhamento e (4) manutenção do vínculo com a APS.

O procedimento utilizado para o agrupamento corresponde à análise de *cluster* TwoStep. O algoritmo da análise TwoStep utiliza a medida de verossimilhança logarítmica, calcula as probabilidades de associação aos *clusters* com base numa ou mais distribuições de probabilidade. Apresenta dois passos, sendo que, no primeiro, os dados são analisados um por um, e grupos homogêneos são formados tendo em conta a medida de distância. Um conjunto de estatísticas que sumarizam a informação sobre o grupo formado é associado a cada grupo . No segundo passo, os grupos obtidos são tratados como observações individuais e um algoritmo hierárquico é utilizado para criar o agrupamento. O objetivo do algoritmo do agrupamento é maximizar a probabilidade global ou a verossimilhança dos dados, observando os agrupamentos finais ^12^.

A medida de distância de verossimilhança utilizada assume que as variáveis contínuas apresentam distribuição normal e categóricas multinomiais, baseada no decréscimo da função de verossimilhança que resulta da união de dois grupos. A medida de silhueta fornece a qualidade do agrupamento. Essa medida interpreta e valida a consistência dentro do agrupamento de dados, sendo calculada com qualquer medida métrica de distância. O valor da silhueta mede quão similar um objeto é ao seu próprio *cluster* (coesão) em comparação com os outros (separação). A silhueta varia de -1 a +1, o valor alto indica que o objeto está bem ajustado ao seu próprio *cluster* e mal ajustado aos *clusters* vizinhos. Assim sendo, entende-se que a medida de qualidade > 0,3, obtida na *clusterização* realizada neste estudo, reflete um modelo satisfatório, seguindo referência do software utilizado SPSS Statistics 29.0.0 (https://www.ibm.com/).

Para identificar associações entre os *clusters* gerados e as variáveis independentes, foi utilizado o teste qui-quadrado. A hipótese nula era de que as variáveis não estavam associadas e a hipótese alternativa era de que as variáveis estavam associadas. A significância estatística foi definida como um valor de p < 0,05. Os cálculos foram realizados com base nos dados válidos, dados faltantes (*missing*) foram excluídos da análise.

### Aspectos éticos

O estudo foi aprovado pelo Comitê de Ética da Faculdade de Ciências Médicas, Universidade Estadual de Campinas (parecer nº 3.793.771), seguindo as normas e diretrizes brasileiras para pesquisas envolvendo seres humanos da *Resolução nº 466/2012* do Conselho Nacional de Saúde ^13^. Os princípios éticos foram assegurados pela garantia do direito de não participação na pesquisa desde o primeiro contato telefônico; anonimato; e adoção do Termo de Consentimento Livre e Esclarecido (TCLE), o qual foi lido em voz alta pelo aplicador do questionário na presença do usuário que, após esclarecer suas dúvidas, assinou o documento. As *Diretrizes para Relato de Estudos Observacionais em Epidemiologia* (Declaração STROBE; https://www.strobe-statement.org/) foram adotadas neste estudo.

## Resultados

Este estudo contou com a participação de 341 usuários. Entre esses, 33,7% (n = 115) eram vinculados ao CAPS II, 34% (n = 116) ao CAPS AD e 32,3% ao ambulatório de especialidades. A caracterização dos participantes segundo o perfil sociodemográfico e diagnóstico é apresentada na Tabela 1.

**Tabela 1:** Caracterização dos participantes do estudo segundo perfil sociodemográfico e diagnóstico (n = 341). São Paulo, Brasil (2023).

Características	n	% (IC95%)
Sexo		
Feminino	165	48,4 (43,1-53,7)
Masculino	176	51,6 (46,3-56,9)
Cor		
Branca	190	55,7 (50,4-60,9)
Parda	119	34,9 (30,0-40,1)
Preta	32	9,4 (6,7-12,9)
Idade (anos)		
18-30	44	12,9 (9,5-16,9)
31-45	113	33,1 (28,3-38,3)
46-60	124	36,4 (31,4-41,6)
61 ou mais	60	17,6 (13,9-21,9)
Escolaridade (anos de estudo)		
0-4	65	19,1 (15,2-23,6)
5-8	144	42,2 (31,1-47,5)
9 ou mais	132	38,7 (33,7-44,0)
Trabalho remunerado		
Não	264	77,4 (73,6-82,3)
Sim	77	22,6 (18,5-27,3)
Renda *per capita* * (salários mínimos)		
Até ½	80	24,8 (20,4-29,7)
½-1	155	48,0 (42,6-53,4)
Mais de 1	88	27,2 (22,7-32,3)
Diagnóstico		
Transtornos afetivos e neuróticos	122	38,8 (30,9-41,0)
Transtornos psicóticos	74	21,7 (17,6-26,4)
Uso problemático de substâncias psicoativas	83	24,3 (20,1-29,2)
Ainda não diagnosticado	62	18,2 (14,4-22,6)

IC95%: intervalo de 95% de confiança.

* A variável renda *per capita* apresentou 18 dados faltantes.

### Caracterização dos *clusters* em relação aos itinerários terapêuticos

Com base nas variáveis selecionadas, foi possível identificar cinco agrupamentos (*clusters*) com elevada homogeneidade interna e heterogeneidade entre si. O tamanho de cada *cluster* variou entre 44 e 101 usuários, a distribuição dos mesmos em relação às suas características pode ser observada na Tabela 2.

**Tabela 2:** Caracterização dos agrupamentos (*clusters*) identificados de acordo com o itinerário terapêutico. São Paulo, Brasil (2023).

*Cluster*	Identificação	Primeiro atendimento	Origem do encaminhamento	Vínculo com a APS
1 (12,9%; n = 44)	Autodiagnóstico (54,4%; n = 24)	APS (65,9%; n = 29)	APS (100,0%; n = 44)	Não (100,0%; n = 44)
2 (22,3%; n = 76)	Situação de crise (100,0%; n = 76)	CAPS II ou CAPS AD (34,2%; n = 26)	Ambulatório de especialidades (56,6%; n = 43)	Não (100,0%; n = 76)
3 (29,6%; n = 101)	Autodiagnóstico (63,4%; n = 64)	CAPS II ou CAPS AD (41,6%; n = 72)	Demanda espontânea (71,3%; n = 72)	Não (86,1%; n = 87)
4 (13,2%; n = 45)	Situação de crise (62,2%; n = 27)	Serviço privado (57,8%; n = 26)	Hospitalar/Emergência (42,2%; n = 18)	Não (95,6%; n = 43)
5 (22,0%; n = 75)	Consulta geral (60,0%; n = 45)	Ambulatório de especialidades (62,7%; n = 47)	APS (33,3%; n = 25)	Não (88,0%; n = 66)

APS: atenção primária à saúde; CAPS II: Centro de Atenção Psicossocial II; CAPS AD: Centro de Atenção Psicossocial - Álcool e Drogas.

O maior *cluster* observado na amostra foi o número três, composto por usuários que majoritariamente tiveram seu problema de saúde mental identificado por meio de autodiagnóstico, primeiro atendimento em saúde mental já em um serviço especializado do tipo CAPS II ou CAPS AD e foram acolhidos no serviço atual por demanda espontânea.

Os *clusters* 2 e 5 apresentaram tamanhos similares, sendo o primeiro constituído por usuários que majoritariamente tiveram seu problema de saúde mental identificado em uma situação de crise, primeiro atendimento em CAPS II ou CAPS AD e foram referenciados para o serviço atual pelo ambulatório de especialidades. Já no *cluster* 5, os usuários foram aqueles que majoritariamente tiveram seu problema de saúde mental identificado em uma consulta por queixas gerais, atendidos inicialmente no ambulatório de especialidades e referenciados para o serviço atual pela APS.

Em menor tamanho dentro da amostra, e compartilhando um número semelhante de usuários, o *cluster* 1 foi composto por usuários que majoritariamente tiveram seu problema de saúde mental identificado por meio de autodiagnóstico, primeiro atendimento na APS e encaminhamento realizado também pela APS. Já o *cluster* 4 foi composto por usuários que majoritariamente tiveram seu problema identificado em uma situação de crise, foram atendidos inicialmente em um serviço privado e tiveram seu encaminhamento realizado para o atual por serviços hospitalares ou de emergência.

Nos cinco *clusters*, a maioria dos usuários não mantinha vínculo com a APS, caracterizado, neste estudo, pelo recebimento de qualquer atendimento nos seis meses que antecederam a coleta de dados.

### Comparação entre os *clusters* de acordo com as características dos usuários

Na Tabela 3, são apresentadas as características sociodemográficas e de diagnóstico dos usuários incluídos no estudo de acordo com o cluster a que pertencem.

Foram observadas diferenças estatisticamente significativas entre os *clusters* no que se refere ao sexo e ao diagnóstico dos participantes. Enquanto os *clusters* 2 e 3 foram caracterizados por uma população majoritariamente masculina, nos *clusters* 1, 4 e 5, houve predomínio de indivíduos do sexo feminino.

Quanto ao diagnóstico, os *clusters* 1, 3 e 5 tiveram maior proporção de indivíduos com transtornos afetivos e neuróticos, havendo destaque de uma proporção significativa de pessoas que faziam uso problemático de substâncias psicoativas no *cluster* 3. Entretanto, foi no *cluster* 4 que houve maior proporção de usuários com esse diagnóstico, sendo o mais frequente desse agrupamento. Já no *cluster* 2, a maior proporção dos usuários apresentava diagnóstico de transtornos psicóticos.

**Tabela 3:** Caracterização dos agrupamentos (*clusters*) identificados de acordo com as características sociodemográficas e diagnóstico. São Paulo, Brasil (2023).

Características	***Cluster* 1**		***Cluster* 2**		***Cluster* 3**		***Cluster* 4**		***Cluster* 5**	
	n	%	n	%	n	%	n	%	n	%
Sexo *										
Masculino	17	38.64	43	56.58	63	62.38	21	46.67	32	42.67
Feminino	27	61.36	33	43.42	28	37.62	24	53.33	43	57.33
Cor										
Branca	24	54.55	39	51.32	57	56.44	27	60.00	43	57.33
Preta	3	6.82	8	10.53	8	7.92	6	13.33	7	9.33
Parda	17	38.64	29	38.16	36	35.64	12	26.67	25	33.33
Idade (anos)										
18-30	8	18.18	8	10.53	14	13.86	4	8.89	10	13.33
31-45	18	40.91	34	44.74	29	28.71	13	28.89	19	25.33
46-60	9	20.45	28	36.84	37	36.63	19	42.22	31	41.33
61 ou mais	9	20.45	6	7.89	21	20.79	9	20.00	15	20.00
Escolaridade (anos de estudo)										
0-4	5	11.36	20	26.32	14	13.86	11	24.44	15	20.00
5-8	22	50.00	30	39.47	45	44.55	14	31.11	33	44.00
9 ou mais	17	38.64	26	34.21	42	41.58	20	44.44	27	36.00
Renda *per capita* ** (salários mínimos)										
Até ½	9	21.95	25	34.25	12	12.90	13	29.55	21	29.17
½-1	25	60.98	31	42.47	51	54.84	20	45.45	28	38.89
Mais de 1	7	17.07	17	23.29	30	32.26	11	25.00	23	31.94
Diagnóstico *										
Transtornos afetivos e neuróticos	21	47.73	17	22.37	35	34.65	9	20.00	40	53.33
Transtornos psicóticos	6	13.64	30	39.47	8	7.92	13	28.89	17	22.67
Uso problemático de substâncias psicoativas	9	20.45	20	26.32	29	28.71	17	37.78	8	10.67
Ainda não diagnosticado	8	18.18	9	11.84	29	28.71	6	13.33	10	13.33

* Valor de p < 0,05;

** A variável renda *per capita* apresentou 18 dados faltantes.

## Discussão

No que se refere à avaliação dos serviços de saúde mental, os estudos brasileiros são marcados por uma tradição na avaliação particular de cada uma das modalidades de serviços que compõem a RAPS, especialmente dos serviços do tipo CAPS ^6^, sendo ainda incipientes os estudos acerca do itinerário terapêutico que buscam relacionar aspectos como o acesso e a utilização dos serviços ^9^.

Como importante ponto de discussão, cabe destacar que o maior cluster encontrado em nosso estudo dizia respeito a pessoas que majoritariamente tiveram como primeiro ponto de contato na RAPS os serviços especializados, a partir de demanda espontânea. Nesse contexto, é pertinente pontuar que o atual funcionamento da RAPS brasileira foi delineado para contemplar o acesso direto aos serviços de saúde mental, especialmente porque o acesso direto pode ser um caminho necessário para o acolhimento imediato em situações agudas ^14^. Contudo, ao considerar a alta proporção de pessoas com transtornos afetivos e neuróticos, bem como os indivíduos sem diagnóstico atribuído nesse agrupamento, pode-se supor que ele representa uma “quebra dos fluxos” de encaminhamento existentes na RAPS.

Em um sistema escalonado como o SUS, há uma expectativa de que o usuário acesse a rede preferencialmente pela APS, que assumiria a coordenação do cuidado e, quando necessário, faria o encaminhamento para o serviço especializado ^15^. Essa função *gatekeeper* é defendida internacionalmente como uma boa prática em saúde mental, por sua potencialidade em garantir altas taxas de detecção precoce e intervenção a nível local, sem sobrecarga dos serviços especializados com demandas que não exijam esse nível de complexidade ^10^. Contudo, a discussão acerca das consequências desse modelo de cuidado têm ganhado corpo ^16^. Aspectos como a baixa capacidade de manejo na APS, somada à burocratização dos fluxos de encaminhamento, têm exacerbado a fila de espera para a utilização dos serviços especializados ^17^. Dessa forma, a existência de múltiplas formas de entrada pode estar modulando a experiência do usuário na rede, que irá buscar a forma mais fácil de acessar o cuidado que precisa, no caso, “batendo à porta” do serviço especializado.

Embora seja um movimento legítimo por parte do usuário, os impactos para organização da RAPS beiram o iníquo. Um estudo recente demonstrou que, em comparação com os usuários absorvidos por demanda espontânea, aqueles referenciados pela APS eram os que tinham menores chances de serem atendidos em tempo oportuno nos serviços especializados ^6^. Dessa forma, pontua-se que a revisão do fluxo de encaminhamentos e as formas de acesso aos serviços da RAPS é um nó crítico que precisa ser urgentemente endereçado pelos tomadores de decisão em nível nacional.

Apesar de o acesso ao serviço especializado ser considerado como um ponto importante do itinerário terapêutico dos usuários ^18^, pois em muitos casos a tecnologia de cuidado oferecida pela APS não é suficiente para a demanda de sofrimento psíquico apresentada pelo usuário, nosso estudo evidenciou que nem sempre ele consiste no ponto de chegada para o tratamento. Essa perspectiva é ilustrada pelos *clusters* 2 e 5; no primeiro, a partir da perspectiva de que o serviço especializado que atendeu inicialmente o caso não foi o mesmo que encaminhou para o serviço atual; e, no segundo, por ter sido a APS que encaminhou o caso para o serviço atual, mesmo após atendimento inicial no ambulatório de especialidades.

O agrupamento caracterizado por usuários que foram encaminhados em sua totalidade pela APS foi o *cluster* 1, caracterizado majoritariamente por usuários que autodiagnosticaram seu problema de saúde mental e procuraram atendimento. Embora esse seja o fluxo antecipado mais esperado ^15^, esse *cluster* correspondeu a apenas 12,9% da amostra, indicando a baixa participação da APS na ordenação do cuidado em rede e, talvez, pouco conhecimento das pessoas que necessitam de cuidados em saúde mental.

A baixa participação da APS em pontos importantes do itinerários terapêuticos dos usuários é um aspecto que merece atenção. Esse aspecto tem sido entendido como um dos principais fatores que contribuem para a superlotação dos serviços especializados, provocando excesso de demandas de baixa complexidade e impossibilitando uma intensidade adequada de atendimento aos casos mais graves ^19^ ^,^ ^20^ ^,^ ^21^ ^,^ ^22^.

Além da superlotação dos serviços especializados, a falta de estratégias para aumentar a capacidade da APS de identificar e intervir nos casos de maneira precoce tem contribuído para o agravamento dos casos, que passam a ser absorvidos pela RAPS somente após uma situação de crise ^14^.

É prioritário que pessoas nessas condições recebam acolhimento e o primeiro atendimento no CAPS. No entanto, no contexto estudado, mesmo que isso ocorra, o atendimento é intermediado pelo ambulatório de especialidades, revelando uma certa função de articulador da rede ainda desempenhada por esse serviço. Há uma escassez de informações na literatura sobre o acolhimento em crises, destacando-se uma lacuna significativa nas evidências sobre os arranjos adotados para integrar serviços comunitários, de emergência e hospitalares na continuidade do cuidado. Essa falta de dados aponta para uma necessidade urgente de maiores investimentos na construção dessas relações ^10^.

Não vamos abordar toda a complexidade envolvida em uma situação de crise e suas diversas dimensões - subjetivas, familiares, sociais e culturais -; porém, é crucial enfatizar a importância de uma análise cuidadosa e a implementação de boas práticas no acolhimento durante crises de saúde mental ^23^. Isso é essencial tanto para o manejo imediato e cuidado contínuo quanto para assegurar que os pontos de atendimento de urgência e emergência em saúde mental estejam integrados de maneira eficiente à rede, otimizando o percurso de cuidado e evitando visitas desnecessárias aos serviços e intervenções inadequadas, especialmente em momentos de maior intensidade de sofrimento.

Nosso estudo também aponta para dois agrupamentos cuja identificação do problema só ocorreu a partir de um quadro de crise, havendo destaque para o *cluster* 4, em que o primeiro atendimento foi realizado por um serviço privado, mas o encaminhamento só ocorreu após internação hospitalar ou atendimento em serviço de emergência. Esse agrupamento, que ilustra com clareza as consequências de uma baixa capacidade de intervenção precoce, chama a atenção para a presença dos serviços privados, em nosso estudo representados principalmente pelas comunidades terapêuticas (CT).

Segundo Passos et al. ^24^, a partir de 2011, em meio à crise da Reforma Psiquiátrica brasileira, as CT foram incorporadas no orçamento público, tendo seu incentivo financeiro de custeio destinado aos estados, municípios e ao Distrito Federal, por meio da *Portaria nº 131/2012* ^25^. Contudo, mesmo com financiamento público, muitas CT não operam em conformidade com os princípios do SUS, infringindo, por exemplo, o princípio da universalidade ao restringir o acesso a pessoas em uso de medicação psicotrópica ou por orientação sexual não heteronormativa ^24^. Adicionalmente, destaca-se que as denúncias de maus-tratos e violações de direitos são frequentes nas CT, incluindo internações excessivamente longas, falta de elaboração de projetos terapêuticos singulares e ausência de fluxos consensuados, resultando em um modelo que fragmenta o cuidado ^26^.

Como a oferta de serviços desempenha um papel crucial nos itinerários terapêuticos, a forte presença de CT na região estudada pode influenciar significativamente a escolha dessas instituições como uma opção de busca por ajuda para problemas relacionados ao uso de substâncias psicoativas, especialmente diante da escassez de respostas dos serviços especializados, como os CAPS, ou da própria APS.

Outro ponto relevante abordado neste estudo é a significativa quantidade de usuários cujo primeiro atendimento em saúde mental ocorreu nos atendimentos de emergência, o que indica uma inversão no fluxo idealizado pela lógica do SUS. Historicamente, os ambulatórios têm frequentemente assumido uma função pautada na lógica especialista e biomédica, muitas vezes desvinculada da rede e em contraposição aos princípios da reforma psiquiátrica brasileira. Essa abordagem reducionista contribui para uma resolução inadequada dos casos, perpetuando o ciclo “crise-internação-alta-crise-reinternação” ^27^ e levando à cronicidade dos usuários. É crucial examinar cuidadosamente o papel desempenhado pelos atendimentos de emergência na rede de saúde do município em questão, assim como em outras RAPS pelo país, e avaliar o impacto que esse *modus operandi* pode ter na assistência à saúde da população. Isso é essencial para evitar práticas iatrogênicas que fragmentam o cuidado dentro da rede.

Por fim, cabe pontuar como característica transversal a todos os agrupamentos observados a não continuidade do vínculo com a APS após a entrada no serviço especializado atual. A proporção dessa característica entre os *clusters* variou de 86,1% a 100%, evidenciando que, mesmo quando o usuário tem o primeiro atendimento na APS ou é encaminhado por ela, há uma baixa continuidade do cuidado.

Essa é uma perspectiva preocupante, uma vez que o fator obesogênico de certas medicações, somado ao estilo de vida não saudável prevalente nessa população, tem resultado em altas taxas de doenças cardiovasculares, diabetes e outras síndromes metabólicas. Essas condições contribuem significativamente para uma taxa de mortalidade duas a três vezes maior entre pessoas com transtornos mentais ^28^. Nesse contexto, nossos resultados enfatizam a importância não apenas de aprimorar a integração entre a atenção primária e os serviços especializados para garantir acesso oportuno ao cuidado em saúde mental, mas também de desenvolver estratégias de contrarreferenciamento dos usuários e incentivar a continuidade do vínculo com esses pontos de atenção ^6^.

Ressalta-se que algumas limitações devem ser consideradas na interpretação de nossos resultados. Algumas variáveis foram medidas de forma retrospectiva, estando sujeitas a viés de memória. Destaca-se, também, que este estudo recrutou usuários que tiveram acesso e permaneceram vinculados aos serviços especializados, não considerando aqueles que não conseguiram acesso ou abandonaram o tratamento. Dessa forma, nossos resultados não são representativos de toda população com transtornos mentais e suas experiências na RAPS estudada.

## Conclusão

Este estudo evidencia a complexidade e os desafios enfrentados pela RAPS no Brasil em um município paulista de médio porte, particularmente em relação ao acesso e à utilização dos serviços de saúde mental. Nossos achados destacam a prevalência do acesso direto aos serviços especializados, muitas vezes por demanda espontânea, o que sugere uma ruptura no fluxo de encaminhamento esperado pela APS. Essa situação reflete tanto uma baixa capacidade de manejo de casos na APS quanto a burocratização dos fluxos de encaminhamento, resultando em superlotação dos serviços especializados e na iniquidade no atendimento.

A baixa participação da APS no itinerário terapêutico dos usuários aponta para uma necessidade urgente de fortalecer sua capacidade de identificar e intervir precocemente em casos de sofrimento psíquico. A dependência de serviços privados, como as comunidades terapêuticas, e a predominância de atendimentos iniciais em serviços de emergência revelam lacunas significativas na continuidade e na coordenação do cuidado, além de potenciais violações dos princípios do SUS.

É crucial que os tomadores de decisão invistam na revisão dos fluxos de encaminhamento e na integração dos serviços de saúde mental, com foco no fortalecimento da APS como coordenadora do cuidado. Além disso, é necessário desenvolver estratégias eficazes de contrarreferenciamento e garantir a continuidade do vínculo dos usuários com a APS, promovendo um cuidado integral e longitudinal.
